# Bone marrow necrosis related to paracoccidioidomycosis: the first eight cases identified at autopsy

**DOI:** 10.1111/j.1365-2559.2009.03245.x

**Published:** 2009-03

**Authors:** Lucilene S R Resende, Rinaldo P Mendes, Maura M Bacchi, Sílvio A Marques, Benedito Barraviera, Lenice R Souza, Domingos A Meira, Lígia Niéro-Melo

**Affiliations:** 1 Haematology Service of Clinical Department, Botucatu Medical School, São Paulo State University, Botucatu, SPBrazil; 2 Departments of Tropical Diseases and Image Diagnosis, Botucatu Medical School, São Paulo State University, Botucatu, SPBrazil; 3 Departments of Pathology, Botucatu Medical School, São Paulo State University, Botucatu, SPBrazil; 4 Dermatology and Radiotherapy, Botucatu Medical School, São Paulo State University, Botucatu, SPBrazil

**Keywords:** bone marrow, bone marrow necrosis, coagulation necrosis, osteonecrosis, paracoccidioidomycosis

## Abstract

**Aims:**

To report the first eight bone marrow necrosis (BMN) cases related to paracoccidioidomycosis (PCM) from patient autopsies with well-documented bone marrow (BM) histology and cytology.

**Methods and results:**

A retrospective evaluation was performed on BM specimens from eight autopsied patients from Botucatu University Hospital with PCM-related BMN. Relevant BMN literature was searched and analysed.

**Conclusions:**

All eight patients had acute PCM. Six had histological only (biopsies) and two cytological only (smears) specimens. Five biopsy specimens revealed severe and one mild coagulation patterned necrotic areas. Five had osteonecrosis. The cytological specimens also showed typical BMN patterns. *Paracoccidioides brasiliensis* yeast forms were visible within necrotic areas in all cases.

## Introduction

Bone marrow necrosis (BMN) can be regarded as a clinicopathological entity when it affects the myeloid parenchyma and medullary stroma, compromising large areas of haematopoietic tissue.^1^ It was described by Wade and Stevenson^2^ in 1941, in a patient with sickle cell disease, and is regarded as a relatively rare finding.^3^,^4^ Although more commonly diagnosed from autopsied than living patients,^5^–^10^ the prevalence of BMN varies from 0.3% to 1% in non-selected populations when considered extensive, from both bone marrow (BM) smears and biopsies.^1^

There are many causes of BMN, but most cases are caused by primary haematological or metastatic malignancies.^1^,^10^–^12^ From an analysis of 240 literature cases with extensive BMN diagnosed during life, Janssens *et al.*^1^ observed that only 9% did not have a causal malignant neoplasm. Non-malignant causes of BMN are varied; they include severe infections caused by bacteria, viruses and fungi.^1^,^12^–^16^

Paracoccidioidomycosis (PCM) is not a routinely described cause of BMN. Although it is the most prevalent systemic mycosis in Latin America, only three previously published papers have exclusively reported it in BM from living patients.^12^,^17^,^18^ Two only superficially cited necrosis in BM smears, describing findings as ‘purulent and necrotic BM sample’^17^ and ‘BM showing cellular necrosis’.^18^ The third paper, published by our PCM research group, was on infiltrative myelopathy by PCM in living patients.^12^ BMN was observed in trephine biopsy specimens from four of those patients, being extensive in just one.

The aim of this study was to report the first eight BMN cases related to PCM identified at autopsy with well-documented BM histology and cytology.

## Materials and methods

A retrospective evaluation was performed on histological or cytological BM specimens obtained from eight autopsied patients with BMN related to PCM. Five of these patients were admitted to the Tropical Diseases Ward, two to the Paediatric ward and one to the Dermatology ward of the Botucatu Medical School University Hospital, São Paulo State University. PCM diagnosis was established during life or at autopsy by identifying typical *Paracoccidioides brasiliensis* yeast forms from different tissue samples. Bone marrow PCM involvement and BMN were both confirmed in all patients at the hospital’s Pathology Service during autopsies. BM PCM involvement was defined as the presence of the *P. brasiliensis* in BM-obtained specimens associated with a variable degree of granulomatous reaction. Coagulation-type BMN was characterized as single or multiple areas of variable extent presenting ghosts of many dead haematopoietic cells within an increased eosinophilic granular stroma in histological samples stained by haematoxylin and eosin (H&E). Bone trabeculae could also be affected.^19^ In smear specimens prepared with Giemsa and/or Shorr stains, BMN was defined by the presence of typical necrotic haematopoietic cells with a smudgy appearance that had blurred the usually crisp nuclear and cytoplasmic staining. A reactive smooth homogeneous background protein could be present.^19^ Finally, attributing the aetiology of BMN to *P. brasiliensis* infection was as a result of observing fungal yeast forms within necrotic areas with any of the above cited stains and/or with Gomori–Grocott stain (GG).

From biopsy specimens, BMN was semiquantitatively graded according its extent, as described by Maisel *et al.*^20^ These criteria classify BMN as Grade I (mild) when the necrotic foci occupy one high-power field (HPF) of 40× or less, or when combined necrotic areas occupy <20% of the entire BM specimen. Grade II necrosis (moderate) is characterized by necrotic foci occupying more than one HPF but less than one 10× field, or when the combined necrotic areas occupy between 20% and 50% of the biopsy specimen. Grade III necrosis (severe) is defined by large foci of necrosis occupying >50% of the specimen.^20^

Epidemiological data and the clinical PCM forms were obtained from each patient’s medical records and analysed. Relevant BMN literature was also sourced and analysed.

This study was approved by the Research Ethics Committee of Botucatu Medical School–São Paulo State University.

## Results

**Figure 1:**
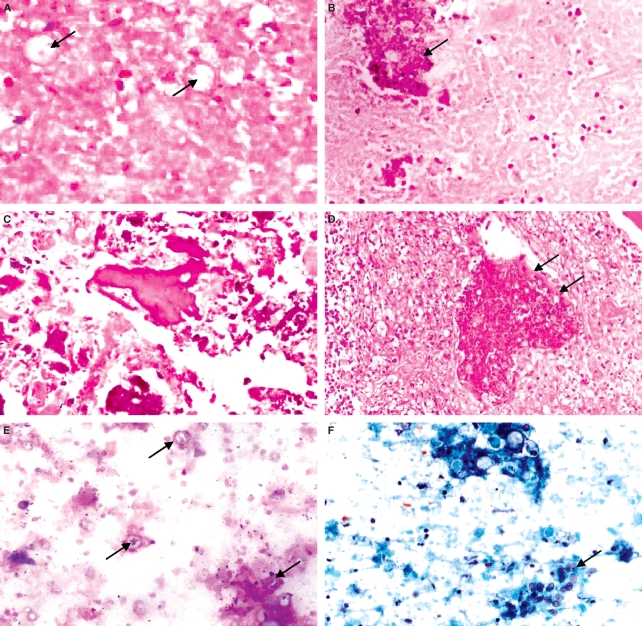
**A**, Case 1: Severe coagulative bone marrow necrosis (BMN) in an AIDS patient. Fungal cells can be observed within the ghosts of the dead hematopoietic cells (arrows). Bone marrow biopsy (BMB); H&E. **B**, Case 2: Extensive coagulative BMN. Bone trabecula was also affected (arrow). BMB; H&E. **C**, Case 5: Disarrangement of bone marrow architecture due to both parenchymal and trabecular severe BMN. BMB; H&E. **D**, Case 6: Necrotic bone trabecula in an extensive area of granulomatous reaction presenting numerous *Paracoccidioides brasiliensis*. Remnant of non-necrotic bone can be seen on its upper border (arrows). BMB; H&E. **E**, Case 7: Necrotic hematopoietic cells with a smudgy appearance. A stained smooth homogeneus background precipitate is present. Fungal cells can be seen (arrows). Aspirated smear; Giemsa. **F**, Case 8: Typical coagulative necrosis on aspirated smear. An agglomerate of histiocyte attempts to form a giant cell (arrow). Several *P. brasiliensis* are also present. Aspirated smear; Shorr.

Table 1 summarizes the main epidemiological data and BM findings of the eight patients. All were Brazilian from São Paulo State and aged between 4 and 23 years; three were male and five female; seven were white and one indigenous. Six lived in rural areas or were rural workers. All eight patients had acute PCM, seven of whom had PCM diagnosed during their lifetime and received specific treatment. One patient (case 8) only had the diagnosis of PCM confirmed at autopsy. Another (case 1) had a simultaneous diagnosis of acquired immunodeficiency syndrome (AIDS). Six only had histological (biopsies) and two only had cytological (smears) BM specimens. The six BM biopsy specimens were stained with H&E. Five revealed severe Grade III (Figure 1A,B) and one revealed mild Grade I coagulation pattern necrosis. Osteonecrosis was observed in five (Figure 1C,D). A cytological specimen from one patient (case 7) was stained with Giemsa’s solution (Figure 1E) and in case 8 by both Giemsa’s and Shorr’s solutions (Figure 1F). All eight cases revealed *P. brasiliensis* yeast forms within the necrotic areas. Four cases were also stained by GG for this purpose.

**Table 1:** Epidemiological data and bone marrow findings from the eight study patients

Cases	Gender	Age (years)	Race	BM biopsy findings	% BMN (BM biopsy)	BM smear findings	Stains
1*	Male	21	White	Coagulation; osteonecrosis	>50	NP	H&E
2	Female	19	White	Coagulation; osteonecrosis	>50	NP	H&E, GG
3	Female	23	White	Coagulation; osteonecrosis	>50	NP	H&E
4	Female	9	Indigenous	Coagulation	>50	NP	H&E, GG
5	Female	18	White	Coagulation; osteonecrosis	>50	NP	H&E, GG
6	Male	6	White	Coagulation; osteonecrosis	<20	NP	H&E
7	Male	21	White	NP	NA	Coagulation	Giemsa’s
8†	Female	4	White	NP	NA	Coagulation	Giemsa’s, Shorr’s, GG

BM, bone marrow; BMN, bone marrow necrosis; NP, not performed; H&E, haematoxylin and eosin; GG, Gomori–Grocott; NA, not applicable.

* AIDS.

† PCM diagnosed during autopsy.

## Discussion

BMN is coagulative in type and occurs because of ischaemia from mechanical or humoral factors causing circulatory failure in BM,^12^ due to vascular compression by intramedullary haematological malignancies or metastatic neoplasms, septic or aseptic microembolization, diseases that cause intravascular fibrin deposition, sickle-cell diseases with vascular occlusive attacks, severe anaemia, cardiocirculatory failure, drugs, toxic agents and other conditions.^1^,^4^,^14^,^15^,^19^ Rarely, severe infections, including those of fungal aetiology, can cause BMN.^1^,^10^,^14^–^16^ Although uncommon, PCM can involve BM to a sufficient extent to cause BMN, mainly in the acute or subacute form.^12^ Patients with this form of the disease have an ineffective Th1 immune response against *P. brasiliensis*, with an increased predominance of the Th2 immunological arm, known to be less effective in disease control.^12^ Acute or subacute PCM then disseminates by lymphatic or lympho-haematogenous routes to organs belonging to the mononuclear phagocytic system, such as the lymph nodes, liver, and spleen; it can also reach the BM.^12^ It is possible that fungal emboli, which result in local fungal proliferation, and production of humoral medullar inflammatory factors, promote tissue ischaemia and subsequent BM coagulative necrosis. Bone trabeculae can also be affected, resulting in simultaneous osteonecrosis, as in five of our cases. The role of AIDS in suppressing the cellular immune response could have contributed to the spread of fungal BM in case 1. AIDS can also cause BMN,^1^,^4^ but the large number of fungal cells within medullary necrotic areas strongly suggests that PCM played a greater role in the pathophysiology of this patient’s necrosis.

BMN prognosis is related to underlying disease severity and is generally poor.^1^,^4^,^10^ It can, however, contribute to the death of some patients^1^ as a consequence of cytopenia that share some of the risks. BMN could have played a role in the deaths of our patients, mainly because most of them showed it in a severe form of this finding.

## Abbreviations:

AIDS: acquired immunodeficiency syndrome
BM: bone marrow
BMB: bone marrow biopsy
BMN: bone marrow necrosis
GG: Gomori–Grocott
H&E: haematoxylin and eosin
HPF: high-power field
PCM: paracoccidioidomycosis
